# Synthesis of Cobalt Particles and Investigation of Their Electromagnetic Wave Absorption Characteristics

**DOI:** 10.3390/ma17010200

**Published:** 2023-12-29

**Authors:** Hong Li, Hongyang Li, Bo Sheng, Bing Zheng, Sujun Shi, Qing Cai, Wenqi Xu, Xiuchen Zhao, Ying Liu

**Affiliations:** Beijing Institute of Technology, School of Materials Science and Engineering, Beijing 100081, China; 840808@bit.edu.cn (H.L.);

**Keywords:** electronics packaging technology, cobalt particles, electromagnetic wave absorber, effective absorption bandwidth

## Abstract

As the integration technology for integrated circuit (IC) packaging continues to advance, the issue of electromagnetic interference in IC packaging becomes increasingly prominent. Magnetic materials, acknowledged for their superior electromagnetic absorption capabilities, play a pivotal role in mitigating electromagnetic interference problems. In this study, we employed a liquid-phase reduction method. We prepared three types of cobalt (Co) particles with distinct morphologies. Through variations in the synthesis process conditions, we were able to control the aspect ratio of protrusions on the surface of the Co particles. It was found that the sword-like Co particles exhibit superior electromagnetic wave absorption capabilities, showing a reflection loss value of up to −50.96 dB. Notably, when the coating thickness is only 1.6 mm, the effective absorption bandwidth is extended up to 7.6 GHz. The spatially expansive sword-like Co particles, with their unique structure featuring dipole polarization and interfacial polarization, demonstrated enhanced dielectric and magnetic loss capabilities, concurrently showcasing superior impedance-matching performance.

## 1. Introduction

As electronic products trend towards miniaturization, increased power, and higher frequencies, there is an exigent requirement for high-performance electronic packaging materials in both system-level and 3D packaging. The rapid evolution of 5G communication technology has further propelled the research and development of these materials. In tandem with the advancement of high-power devices, the mitigation of electromagnetic interference among electronic devices has emerged as a focal point of attention [1,2].

In the ever-evolving realm of integrated circuit (IC) packaging technology, extending beyond the scope of Moore’s Law [3,4], the ongoing trajectory plays a pivotal role. This progression, however, exacerbates electromagnetic compatibility (EMC) concerns within IC packaging. The emergence of the 5G era adds another layer of complexity to the electromagnetic environment in communication systems. This introduces a myriad of radiation interference signals that can detrimentally impact the functionality of electronic components [5].

Electromagnetic wave absorbers (EMWAs) assume a crucial role in attenuating and dissipating incident electromagnetic waves (EMW), thereby achieving effective EMW absorption. This capability provides a viable solution for radiation suppression within system-level packaging. The development of EMWAs with characteristics such as lightweight, ultra-wideband functionality and robust absorption is of paramount significance for effectively attenuating EMW [6].

EMWAs predominantly leverage the intrinsic dielectric and magnetic properties of materials to convert electromagnetic energy into thermal energy [7]. Furthermore, these materials find extensive applications in radar stealth coatings, electromagnetic shielding, data protection, microwave darkrooms, and various other fields [8,9,10]. Depending on the form of energy loss in absorption materials, EMWAs can be classified into dielectric absorption materials, magnetic loss absorption materials, and dielectric/magnetic composite absorption materials.

Magnetic materials, primarily composed of magnetic metals such as Fe, Co, and Ni, function as EMW absorption materials [11,12]. These materials are capable of converting the received energy from electromagnetic waves into other forms of energy [13]. Co, being a ferromagnetic metal material, possesses advantages such as a high Curie temperature and thermal stability, making it a subject of extensive research for its EMW loss. Co can provide both magnetic and dielectric losses upon interaction with an electromagnetic wave. In this investigation, we delved into the captivating realm of EMW absorption, examining the performance of three unique Co particles. These particles, each boasting distinctive morphologies, were meticulously crafted using an intriguing liquid-phase reduction method.

## 2. Experiments Methods

### 2.1. Preparation of Co Particles

**Figure 1:**
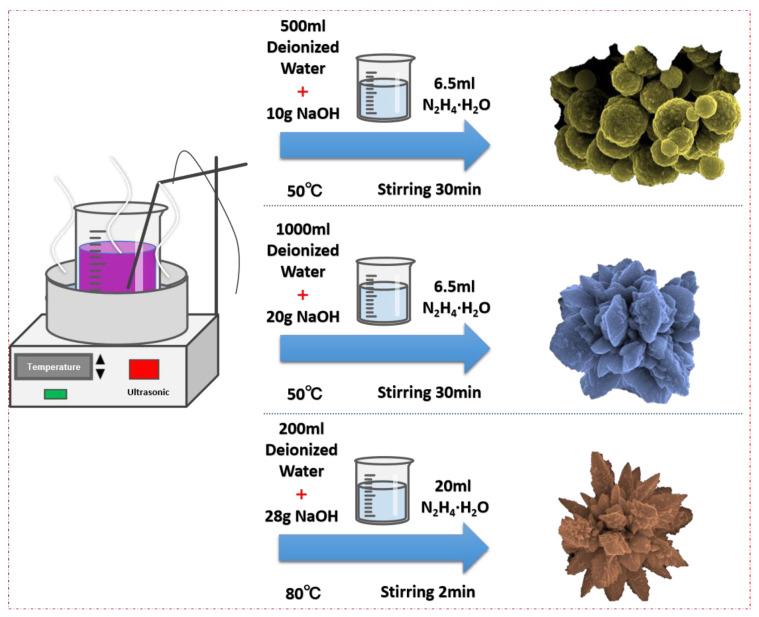
Schematic illustrating the synthesis process of Co particles.

Figure 1 illustrates the process flow for the preparation of spherical Co particles, flower-like Co particles, and sword-like Co particles.

Preparation of spherical Co particles: Weighing 5.2 g of CoSO_4_·7H_2_O (Macklin, Shanghai) and KNaC_4_H_12_O_10_·4H_2_O (Kermel, Tianjin, China) separately, they were dissolved in 500 mL of deionized water. The mixture was allowed to stand for 3 min to induce complexation, followed by the addition of 10 g of NaOH (Macklin, Shanghai, China) to adjust the pH of the reaction solution to greater than 13. Under conditions of mechanical stirring and ultrasonic vibration, the temperature of the reaction solution was maintained at 50 °C using an ultrasonic generator. Subsequently, 6.5 mL of N_2_H_4_·H_2_O (Aladdin, Shanghai, China) was poured into the reaction solution, and the reaction proceeded for 30 min, resulting in the formation of spherical Co particles.

Preparation of flower-like Co particles: The procedure for synthesizing flower-like Co particles mirrored that employed for spherical Co particles. Specifically, 5.2 g each of CoSO_4_·7H_2_O and KNaC_4_H_12_O_10_·4H_2_O were dissolved in 1000 mL of deionized water. To attain a pH surpassing 13, 20 g of NaOH was introduced. The reaction solution’s temperature was held at 50 °C using an ultrasonic generator. Subsequently, 6.5 mL of N_2_H_4_·H_2_O solution was poured into the reaction solution, and the reaction proceeded for 30 min, resulting in the formation of flower-like Co particles.

Preparation of sword-like Co particles: In the preparation of sword-like Co particles, a methodology akin to that utilized for spherical Co particles was implemented. Specifically, 5.2 g of CoSO_4_·7H_2_O and KNaC_4_H_12_O_10_·4H_2_O were dissolved in 200 mL of deionized water. Adjusting the pH of the reaction solution to surpass 13 was achieved by incorporating 28 g of NaOH. Under the conditions of mechanical stirring and ultrasonic vibration, the reaction solution temperature was carefully maintained at 80 °C using an ultrasonic generator. Subsequently, 6.5 mL of N_2_H_4_·H_2_O solution was poured into the reaction solution, and the reaction proceeded for 2 min, resulting in the formation of sword-like Co particles.

### 2.2. Characterization of Co Particles

**SEM:** The morphology of Co particles was observed using the GeminiSEM 300 electron scanning microscopy (SEM) from Carl Zeiss Microscopy GmbH (Jena, Germany), with an acceleration voltage of 15 kV.

**X-ray diffraction (XRD):** The crystal structure of Co particles was determined using the PANalytical X’pert3 (PANalytical, Alemlo, The Netherlands), X-ray diffractometer with Cu Kα radiation (λ = 1.541874 Å). The instrument operated at 40 kV and 40 mA, with a scanning step size of 0.02° and a scanning speed of 1°/min.

**Static magnetic property:** The static magnetic properties of Co particles, including saturation magnetization, coercivity, and remanence, were measured using the Lake Shore 7410 (Lake Shore, Columbus, OH, USA) vibrating sample magnetometer (VSM).

**Vector network analyzer:** Under heating conditions, Co particles were uniformly mixed with paraffin (mass ratio of 7:3) and pressed into smooth, flat, circular ring-like samples using a mold. The inner diameter of the ring was 3.00 mm, the outer diameter was 7.00 mm, and the thickness of the ring face was approximately 2 mm. The HP8722ESS vector network analyzer (Agilent, Santa Clara, CA, USA), based on the transmission line theory, was employed. The testing frequency ranged from 2 to 18 GHz, measuring the complex permittivity (*ε_r_*) and complex permeability (*μ_r_*) of the Co particle/paraffin sample [14]:

(1) $$\varepsilon_{r}=\varepsilon^{\prime }-j\varepsilon \prime \prime$$

(2) $$\mu_{r}=\mu^{\prime }-j\mu \prime \prime$$

The electromagnetic parameters *μ*′ and *μ*″, as well as *ε*′ and *ε*″, represent the real and imaginary parts of the complex magnetic permeability and complex permittivity, respectively.

The reflection loss (*RL*) and impedance matching (*Z*) of the sample were calculated using the following formulas [15,16,17]:

(3) $$RL\left(dB\right)=20log|(Z_{in}-Z_{0})/\;(Z_{in}+Z_{0})|$$

(4) $$Z=\left|\frac{Z_{in}}{Z_{0}}\right|=\sqrt{\left|\frac{\mu_{r}}{\varepsilon_{r}}\right|}\tanh\left[j(2\pi fd/c)\sqrt{\mu_{r}\varepsilon_{r}}\right]$$

where *Z*_0_ stands for the characteristic impedance of free space, while *Z_in_* denotes the characteristic impedance of the absorption material, *c* represents the speed of light in a vacuum, *f* is the frequency of EMW propagation, and *d* is the thickness of the absorption material.

## 3. Results and Discussion

**Figure 2:**
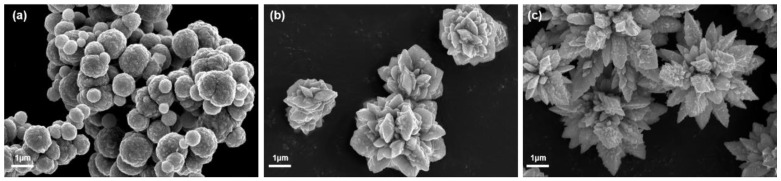
The SEM images of the (**a**) spherical Co particles; (**b**) flower-like Co particles; (**c**) sword-like Co particles.

Figure 2 illustrates SEM images of Co particles, depicting the various shapes achieved through three distinct preparation processes. In Figure 2a, spherical Co particles are observed with a surface adorned with micro-convex protrusions, measuring approximately 500 nm to 1 μm. Figure 2b displays short and robust flower-like Co particles with dimensions around 2–3 μm. Finally, Figure 2c exhibits sharp and pointed sword-like Co particles, with dimensions approximately ranging from 3–5 μm.

The analysis suggests that in the liquid-phase reduction reaction, it is beneficial for the preferential growth of cobalt particles and the diffusion of cobalt atoms to change the reaction conditions. Thus, cobalt particles of different shapes are obtained [18].

**Figure 3:**
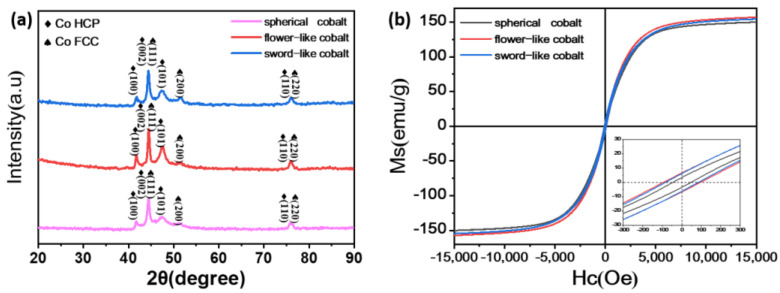
(**a**) XRD patterns of Co particles; (**b**) magnetic hysteresis loops.

Figure 3 displays the XRD patterns of Co particles, revealing a nearly identical crystal structure for the three types. The characteristic diffraction peaks at 2*θ* = 44.216°, 51.522°, and 75.853° align precisely with the positions for FCC-Co’s (PDF: 15-0806) (111), (200), (220) crystal planes. The peaks at 2*θ* = 41.683, 44.762, 47.568 and 75.939° correspond to HCP-Co’s (PDF: 05-0727) (100), (002), (101), and (110) crystal planes, respectively, while FCC-Co’s (111) and (220) crystal planes coincide with HCP-Co’s (002) and (110) crystal planes, respectively. This finding suggests that the three prepared Co particle types simultaneously possess HCP and FCC structures. However, the intensity of the diffraction peaks associated with HCP-Co’s (100) and (101) crystal planes in the flower-like Co particles indicates a higher content of hexagonal close-packed crystal structure, followed by sword-like Co particles, and finally, spherical Co particles.

The hysteresis loop graphs for the three Co particle morphologies, as depicted in Figure 3b, provide values for the saturation magnetization (Ms), coercivity (Hc), and remanence (Br), as summarized in Table 1.

All three Co particle types exhibit high saturation magnetization, beneficial for enhancing complex permeability and, consequently, magnetic loss [19]. However, owing to the mixed crystal structure of FCC and HCP in Co particles, wherein the magnetic crystalline anisotropy of the hexagonal close-packed structure (5.2 × 10^5^ J/m^3^) exceeds that of the face-centered cubic structure (−6.5 × 10^4^ J/m^3^) [20], flower-like Co particles with a higher content of hexagonal close-packed structure exhibit stronger coercivity. This finding aligns with the XRD analysis results. Simultaneously, Hc is influenced by the shape anisotropy of magnetic particles, and the Hc of randomly oriented magnetic particles with a large aspect ratio is determined by the difference in demagnetization factors between the long and short axes. Compared to flower-like Co particles and sword-like Co particles, the demagnetization factor difference of spherical Co particles is relatively small, resulting in a lower Hc for spherical Co particles [21].

**Figure 4:**
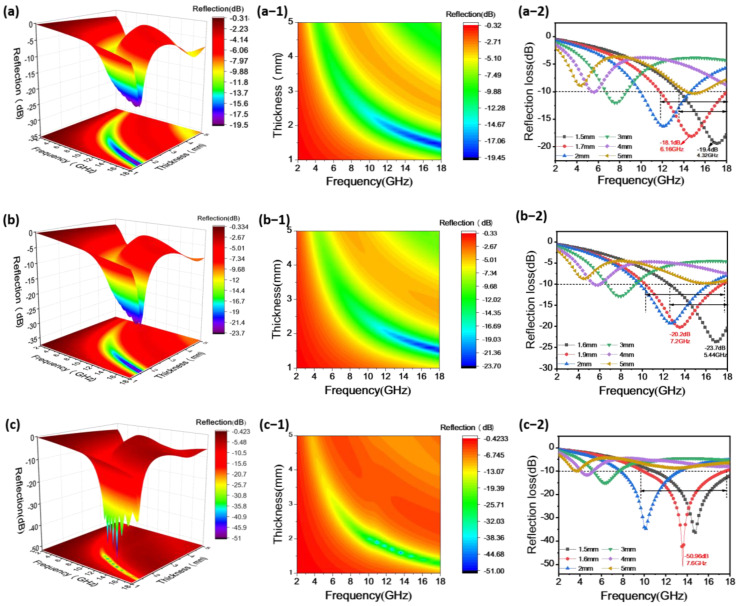
Three-dimensional (3D) RL, 3D projection plots, and the RL values of 70% Co content (**a,a-1,a-2**) spherical Co particles; (**b,b-1,b-2**) flower-like Co particles; and (**c,c-1,c-2**) sword-like Co particles.

The EMW absorption performance of the material can be calculated using transmission line theory based on the material’s electromagnetic parameters to determine the material’s RL. A lower RL value for absorption materials indicates more ideal absorption. Generally, when the RL value of an absorption material is less than −10 dB, it means that the material has absorbed 90% of the incident EMW, demonstrating effective absorption. The effective bandwidth of absorption materials refers to the frequency range corresponding to an RL below −10 dB. Figure 4 depicts the three-dimensional (3D) RL, 3D projection plots, and the RL values between RL, absorption layer thickness, and frequency of Co particles.

In Figure 4(a,a-1,a-2), it is observed that the maximum RL value for spherical Co particles is −19.4 dB, corresponding to a frequency of 17.12 GHz, a coating thickness of 1.5 mm, and an effective absorption bandwidth of 4.32 GHz. However, within the frequency range of 2–18 GHz, when the coating thickness is 1.7 mm, the maximum effective absorption bandwidth of the sample is 6.16 GHz, covering the entire Ku-band (12 GHz–18 GHz). The maximum RL value at this coating thickness is −18.1 dB, corresponding to a frequency of 14.72 GHz.

In Figure 4(b,b-1,b-2), for flower-like Co particles, the maximum RL value is −23.7 dB at a frequency of 16.88 GHz, with a coating thickness of 1.6 mm and an effective absorption bandwidth of 5.44 GHz. However, within the frequency range of 2–18 GHz, when the coating thickness is 1.9 mm, the maximum effective absorption bandwidth of the sample is 7.2 GHz, covering the frequency range of 10.32–17.52 GHz. The maximum RL value at this coating thickness is −20.2 dB, corresponding to a frequency of 13.52 GHz.

In Figure 4(c,c-1,c-2), for sword-like Co particles, the maximum RL value is −50.96 dB at a frequency of 13.6 GHz, with a coating thickness of 1.6 mm and an effective absorption bandwidth of 7.6 GHz. Within the frequency range of 2–18 GHz, the corresponding frequency range is 9.92–17.52 GHz. It is evident from the graph that as the thickness increases, the maximum RL peak shifts to lower frequency bands. Sword-like Co particles exhibit lower RL and the maximum effective absorption bandwidth among the three Co particles.

**Figure 5:**
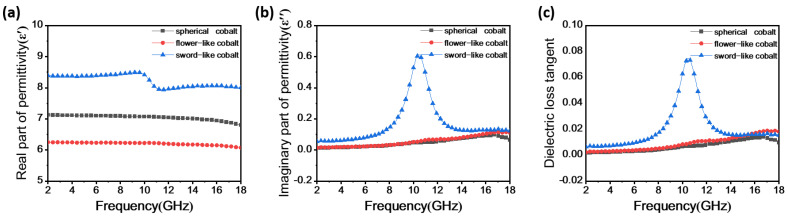

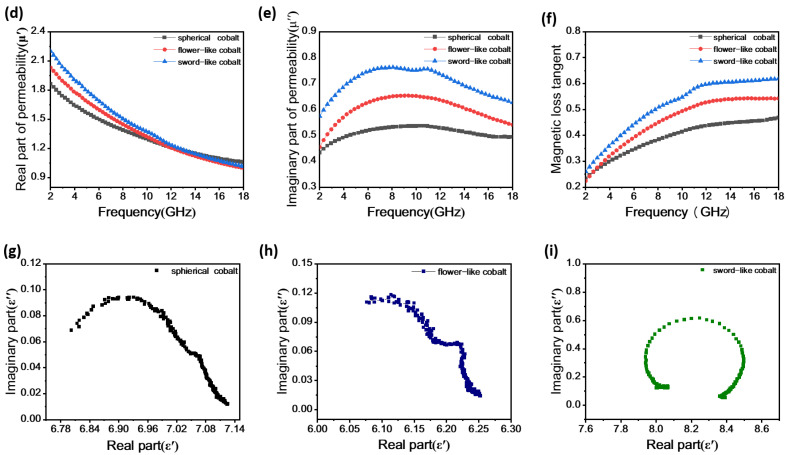
Complex permittivity (**a**,**b**), dielectric loss (**c**), complex permeability (**d**,**e**), magnetic loss (**f**), and Cole–Cole semicircle images (**g**–**i**) of samples.

For a material to demonstrate notable RL, it must possess high complex permittivity and complex permeability within the frequency band of EMW [22,23,24]. Figure 5 illustrates the curves of various electromagnetic parameters for the three different morphologies of Co particles as a function of frequency in the range of 2–18 GHz.

In Figure 5a, it is observed that with increasing frequency, the *ε*′ values of spherical, flower-like, and sword-like Co particles gradually decrease due to the frequency dispersion [25]. The ranges are 7.12 to 6.8, 6.25 to 6.07, and 7.12 to 6.8, respectively. The *ε*′ value of the sword-like Co particles is higher than that of the spherical and flower-like Co particles, with a dielectric resonance peak occurring at 9.68 GHz, while the spherical and flower-like Co particles do not exhibit a dielectric resonance peak.

Figure 5b shows the curves of the imaginary part of the complex permittivity (*ε*″) as a function of frequency. In the range of 2 GHz to 18 GHz, the *ε*″ values of the sword-like Co particles vary from 0.05 to 0.61, higher than the ε″ values of the spherical Co particles (0.012 to 0.097) and the flower-like Co particles (0.014 to 0.118). A strong dielectric resonance peak is observed at 10.4 GHz, lagging behind the peak of *ε*′, indicating that the sword-like Co particles have stronger electrical storage and loss capabilities.

The losses of absorption materials to EMW primarily involve dielectric loss and magnetic loss, which can be represented by the tangent of the dielectric loss angle tan*δ_e_ = ε*″/*ε*′ and the tangent of the magnetic loss angle tan*δ_m_ = μ*″/*μ*′ [26]. Figure 5c depicts the curves of tan*δ_e_* for the three Co particle samples as a function of frequency, consistent with the *ε*″ curves. The tan*δ_e_* curve for the sword-like Co particles exhibits a resonance peak, and within the electromagnetic frequency range of 2–18 GHz, tan*δ_e_* ranges from 0.006 to 0.075, higher than the other two samples, indicating that the sword-like Co particles have the highest dielectric loss. The dielectric loss of absorption materials mainly includes conductivity loss and polarization loss, where polarization loss encompasses ionic polarization, electronic polarization, dipole polarization, and interface polarization. Electronic and ionic polarizations occur over a very short time and typically at high frequencies (103–106 GHz) [27,28]. Therefore, the dielectric loss of Co particle materials is primarily attributed to various dipole polarizations, and the Debye relaxation model is commonly used to verify the dipole polarization of absorption materials [29], i.e., the real part (*ε*′) and imaginary part (*ε*″) of the complex permittivity are related as follows:

(5) $$(\varepsilon \prime -\frac{\varepsilon_{s}+\varepsilon_{\infty }}{2}{)}^{2}+(\varepsilon \prime \prime {)}^{2}=(\frac{\varepsilon_{s}-\varepsilon_{\infty }}{2}{)}^{2}$$

where *ε_s_* indicates static dielectric constant, and *ε_∞_* indicates infinite frequency dielectric constant. It can be observed that the curves of the real part (*ε*′) and imaginary part (*ε*″) of the complex permittivity represent a semicircle, commonly defined as the Cole–Cole semicircle. However, due to various influencing factors, the Cole–Cole semicircle is not a perfect circle [30], and each semicircle corresponds to a Debye polarization relaxation process. The Cole–Cole semicircles for the Co particle samples are shown in Figure 5g–i. It can be noted that the three Co particle samples exhibit two Debye polarization relaxation processes. The analysis suggests that defects are generated during the preparation process, leading to the formation of electric dipoles. The sword-like Co particles, in particular, exhibit more pronounced defects, resulting in a more evident Cole–Cole semispherical ring. Therefore, the two polarization processes are attributed to the orientation polarization of inherent electric dipoles and the interfacial polarization between Co particles and paraffin.

Figure 5d shows the real part (*μ*′) of the complex permeability. It is observed that the *μ*′ values for the three samples show a slight decrease trend. As the frequency increases, the ranges of *μ*′ for spherical, flower-like, and sword-like Co particles are 1.05 to 1.86, 1.00 to 2.03, and 1.01 to 2.2, respectively. Figure 5e presents the imaginary part (*μ*″) of the complex permeability. The *μ*″ values for spherical, flower-like, and sword-like Co particles increase and then decrease with frequency. The ranges are 0.433 to 0.536, 0.455 to 0.654, and 0.573 to 0.763, respectively, with the sword-like Co particles having significantly higher *μ*″ values than the other two samples.

Figure 5f illustrates the curves of tan*δ_m_* for the three Co particle samples as a function of frequency, consistent with the curves of *μ*″ as a function of frequency. In the frequency range of 2–18 GHz, the tan*δ_m_* for the sword-like Co particles ranges from 0.261 to 0.618, higher than that of the spherical and flower-like Co particles.

In summary, the sword-like Co particles exhibit larger dielectric and magnetic losses. However, since the tan*δ_m_* values for the three samples are higher than the tan*δ_e_* values, Co particles primarily dissipate EMW energy through magnetic loss.

The primary cause of EMW losses in Co particles is magnetic losses, primarily originating from hysteresis loss, domain wall resonance, eddy current effects, and natural resonance in magnetic materials. Hysteresis loss occurs irreversibly under higher electromagnetic fields [31]. Domain wall resonance in polycrystalline materials primarily occurs within a lower frequency range (f < 2 GHz). Therefore, both hysteresis loss and domain wall resonance losses can be neglected. If the magnetic losses in the material are solely attributed to the eddy current effect, the relationship is as follows [31]:

(6) $$C_{0}=\mu \prime \prime {\left(\mu \prime \right)}^{-2}f^{-1}=2\pi \mu_{0}\sigma d^{2}/3$$

**Figure 6:**
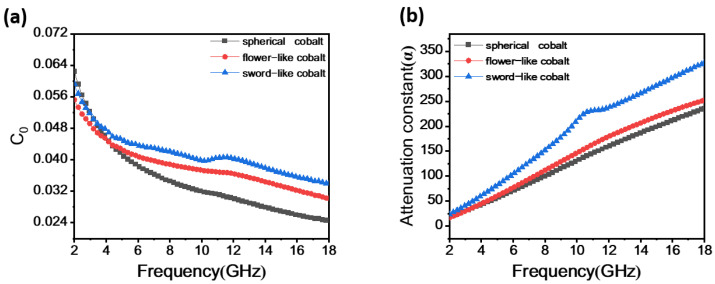
(**a**) *C_0_* curve of Co sample and (**b**) attenuation constant curve.

In Equation (6), where *d* represents the sample thickness, *μ*₀ is the vacuum permeability, *f* is the EMW frequency, and *σ* is the electrical conductivity, the *C*₀ value remains constant without varying with frequency; it is a constant value [32]. However, as illustrated in Figure 6a, it is evident that the *C*₀ values of the three samples decrease with increasing frequency. In the frequency range of 2–5 GHz, the variation is substantial, indicating that magnetic losses within this frequency range are caused by natural resonance. In the 5–18 GHz frequency range, the variation slows down, yet significant fluctuations persist, suggesting that magnetic losses in this frequency range are jointly induced by eddy current losses and natural resonance. The sword-like Co particles exhibit a pronounced resonance peak in the 10–12 GHz range, attributed to exchange resonance-induced magnetic losses.

Simultaneously, the diverse surface morphologies of Co particles significantly influence absorption performance, especially in the case of flower-like and sword-like Co particles, which possess excellent specific surface areas. Their cross-stacking further forms a three-dimensional network, enhancing the absorber’s anisotropy field. With an increase in the aspect ratio of the particle surface length to diameter, magnetic dipole moments are generated at the tips, producing stray magnetic fields. The interaction of these dipoles with incident EMW weakens the stray magnetic field, facilitating the conversion of EMW energy into heat [33,34].

The attenuation characteristics of absorption materials are typically expressed through the attenuation constant *α*, with a higher *α* indicating a greater ability of the absorption material to diminish incident EMW [35,36,37]. In general, the *α* value can be expressed by the following equation:

(7) $$\alpha =\left(\frac{\sqrt{2}\pi f}{c}\right)\times \sqrt{\left(\mu^{\prime \prime }\varepsilon^{\prime \prime }-\mu^{\prime }\varepsilon^{\prime }\right)+\sqrt{({\mu^{\prime \prime }\varepsilon^{\prime \prime }-\mu^{\prime }\varepsilon^{\prime })}^{2}+{\left(\varepsilon^{\prime }\mu^{\prime \prime }+\varepsilon^{\prime \prime }\mu^{\prime }\right)}^{2}}}$$

As depicted in Figure 6b, there is a gradual increase in *α* values with an elevation in frequency. The ranges of *α* values for the spherical Co particles, flower-like Co particles, and sword-like Co particles are 17.75–235.47, 16.81–252.19, and 23.86–327.09, respectively. Within the 2–18 GHz range, the *α* value of the sword-like Co particles surpasses that of the other two particles. Hence, the sword-like Co particles exhibit a superior capability for attenuating EMW.

If there is an impedance mismatch between free space and magnetic materials, strong reflections occur when EMW reaches the surface of the magnetic material. Only a small fraction of EMW can penetrate the material, thereby reducing the material’s absorption of EMW. Conversely, when there is impedance matching between free space and magnetic materials, a greater number of EMW can enter the material, increasing its absorption of EMW [38]. Impedance matching close to 1 indicates that EMW can avoid significant reflection on the material surface and penetrate into the material’s interior.

**Figure 7:**
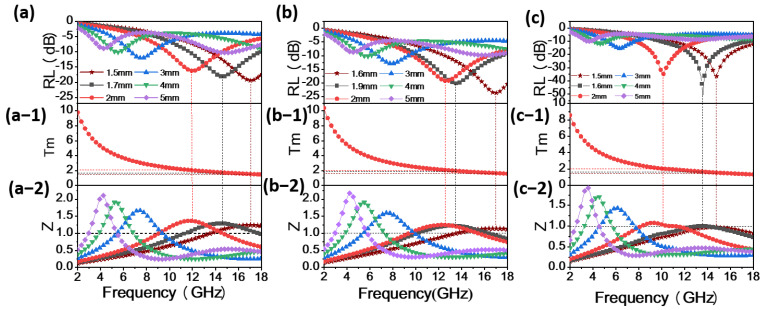
Dependence of the matching thickness (Tm) on frequency under λ/4 and normalized input impedance of (**a,a-1,a-2**) spherical Co, (**b,b-1,b-2**) flower-like Co, and (**c,c-1,c-2**) sword-like Co.

Figure 7 illustrates the relationship curves between the RL, simulated thickness, and impedance matching for the three Co particle samples at the coating thickness corresponding to their maximum RL. It can be observed that with increasing thickness, the peak of the RL shifts towards lower frequencies. This phenomenon can be analyzed using the quarter-wavelength theory [39].

(8) $$T_{m}=\frac{n\lambda }{4}=\frac{nc}{4f_{m}\sqrt{\left|\mu_{r}\right|\left|\varepsilon_{r}\right|}}(n=1,3,5,\ldots )$$

Here, *λ* represents the wavelength of EMW, $T_{m}$ and $f_{m}$ are the thickness and frequency corresponding to the maximum RL, and $\mu_{r}$ and $\varepsilon_{r}$ are the complex permeability and complex permittivity at the corresponding frequency, respectively. Physically, when $T_{m}$ and *f_m_* satisfy the quarter-wavelength matching model, the two reflected waves have a phase difference of 180°, forming a standing wave that dissipates each other, thereby achieving the effect of EMW absorption. As shown in Figure 7(a-1,b-1,c-1), the Tm fit curves of spherical Co, flower-like Co, and sword-like Co were simulated with Formula (8), and the matching thicknesses marked with dashed lines were obtained from the RL curves. It is evident that the experimental results are in complete agreement with the theoretical calculations at the thicknesses of 1.5 mm, 1.6 mm, and 1.6 mm, indicating that the RL curves of spherical Co, flower-like Co, and sword-like Co conformed to the quarter-wavelength matching model [26].

**Figure 8:**
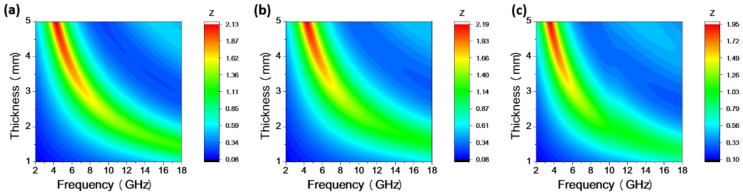
Z values for samples of (**a**) spherical Co, (**b**) flower-like Co, and (**c**) sword-like Co.

Meanwhile, as shown in Figure 7(a-2,b-2,c-2), for the spherical Co particles, flower-like Co particles, and sword-like Co particles, the Z values corresponding to the maximum RL are 1.24, 1.14, and 0.99, respectively. The Z value for the sword-like Co particles is closer to 1, indicating not only the lowest RL at a thickness of 1.6 mm but also excellent impedance-matching performance, allowing for maximum EMW absorption [40]. According to Formula (4), the Z values can be calculated. In Figure 8, the Z values corresponding to thicknesses in the range of 1–5 mm show that the Z value for the sword-like Co particles is closer to 1, indicating superior impedance-matching performance.

## 4. Conclusions

This study employs a liquid-phase reduction method to prepare magnetic Co particles with both FCC and HCP structures. The aspect ratio of protrusions on the surface of Co particles can be controlled by changing the experimental conditions. The experimental results reveal that the Ms of Co particles with different morphologies is similar, but due to the magnetic crystal anisotropy and shape anisotropy of Co particles, flower-like Co particles with more FCC phases and sword-like particles with larger aspect ratios on the particle surface have higher Hc.

The sword-like Co particles demonstrate noticeable dielectric resonance peaks under the testing conditions of 2–18 GHz. They also exhibit typical magnetic particle properties, including natural resonance, exchange resonance, and eddy current loss. Consequently, when compared to spherical Co particles and flower-like Co particles, the sword-like Co particles, with a relatively large aspect ratio, exhibit a maximum RL of −50.96 dB at a frequency of 13.6 GHz. This optimum RL corresponds to a coating thickness of 1.6 mm, with an effective absorption bandwidth of 7.6 GHz within the frequency range of 9.92–17.52 GHz.

The enhanced electromagnetic absorption properties could be ascribed to the unique structure, dipole polarization, interfacial polarization, synergistic effect of dielectric loss and magnetic loss, superior impedance matching, and quarter-wavelength cancellation. Thus, the Co particles with a large aspect ratio could be a promising candidate to develop high-performance microwave absorbers. As EMWAs for IC packaging, testing of the electromagnetic protection performance of chip packaging is currently underway, and the relevant results will be presented in subsequent articles.

## Figures and Tables

**Table 1:** Magnetic properties of Co particles.

Sample	Ms (emu/g)	Br (emu/g)	Hc (Oe)
Spherical Co	150.2	3.3	48.9
Flower-like Co	157.1	6.4	98.1
Sword-like Co	154.6	6.1	85.1

## Data Availability

Data are contained within the article.
